# Magnitude of depression and its associated factors among patients with diabetes mellitus at public hospitals in Southwest Ethiopia, 2021

**DOI:** 10.1038/s41598-022-26330-8

**Published:** 2022-12-22

**Authors:** Habtamu Birhanu, Sabit Zenu, Tadesse Sheleme, Bilisumamulifna Tefera Kefeni

**Affiliations:** 1 Department of Public Health, College of Health Science, Mattu University, Mattu, Ethiopia; 2 Department of Pharmacy, College of Health Science, Mattu University, Mattu, Ethiopia

**Keywords:** Endocrinology, Health care, Neurology

## Abstract

Depression is the third-leading cause of disability measured in terms of disability-adjusted life-years. When depression coexists with diabetes mellitus, it is associated with major health consequences and results in poor health outcomes, decreased quality of life, lost productivity and increased risk of death. The current study aimed to assess the magnitude of depression and its associated factors among adult patients with diabetes mellitus attending follow-up at the public hospitals of Buno Bedele zone, Southwest Ethiopia. A multi-centre cross-sectional study was done among people living with diabetes mellitus at an outpatient clinic of Buno Bedele zone hospitals. The study period was from April to May 2021. A systematic random sampling technique was used to select the study participants. Data were collected using a structured questionnaire. Depression was assessed by the Patient Health Questionnaire-9. Patient Health Questionnaire-9 is a validated tool to assess depression with sensitivity 86% and specificity 67%. The collected data were cleaned, edited, and entered into epi-data version 3.1, and analysed using SPSS version 24. Logistic regression analysis was employed to identify factors associated with depression occurrence. A *p* value of 0.05 was considered statistically significant. A total of 310 study participants were included in this study. Among study participants, 41.6% fulfilled the criteria for depression. Variables significantly associated with depression were female gender [AOR: 2.26, 95% CI (1.30, 3.95)], duration of diabetes greater than 5 years [AOR: 2.68, 95% CI (1.57, 4.56)], poor social support [AOR: 2.46, 95% CI (1.10, 5.49)], moderate social support [AOR: 2.63, 95% CI (1.34, 5.16)], current alcohol consumption [AOR: 3.55, 95% CI (1.20, 10.52)] and previous alcohol consumption [AOR = 2.81, 95% CI (1.40, 5.60)]. According to this study, depression is relatively common among diabetic individuals. Being a female, having diabetes for a long time, having poor social support, using alcohol now and in the past were factors that substantially linked to depression. Healthcare professionals should consider screening for depression using the Patient Health Questionnaire-9 or other validated tools in all diabetic patients, especially in those who are at higher risk.

## Introduction

Globally, depression is the most common psychiatric disorder and the most frequent mental disorder in primary health care. It is a substantial contributor to the global burden of disease^1,2^. It is the third leading cause of disability measured in terms of disability-adjusted life-years in the world^3^ and is expected to be a leading cause of disability around the world and contributes greatly to the global burden of disease by 2030^4^.

In developed countries, the estimated prevalence of major depression was 5.5% and 5.9% in developing countries^5^. People living with diabetes mellitus (DM) have been reported to be more likely to develop depression than non-diabetes people. About 15%-20% of people with diabetes are suffering from depression^6^. A meta-analysis showed that the overall pooled estimated prevalence of depression among diabetes population patients was 39.73% in Ethiopia^7^.

Although the exact cause of depression in people with diabetes is unknown, it is likely complex due to a combination of genetic, physiological, and psychological factors^6^. The biggest increment in depression is expected among people above 60 years of age, who are more likely affected by lifelong diseases^8^. Environmental and social factors affecting self-care have a significant load on patients and their families and this may contribute to depression^9^.

When depression and diabetes are coexist, the patients can be affected by major health consequences like morbidity and mortality^10^. The burden of depression with diabetes is a main public health challenge for developing countries^3^. Depression plus diabetes aggravate clinical outcomes for both diseases, result in poor medication adherence, decrease response to treatment, and increase morbidity and mortality^11^. The coexistence of both diseases also increases the burden of physical illness, functional impairment, and medical costs^12^. Depression forecasts the beginning, progression, and level of disability associated with diabetes. The quality of life for diabetes patients is significantly lower if depression is co-exist^8^.

Despite the efforts in practice, assessing and controlling of depression among patients with diabetes remain a main challenge particularly in developing countries^11^. Depression remains unidentified and untreated in approximately two-thirds of diabetes^13^. Unidentified and untreated depression is related with poor treatment outcomes of being treated chronic diseases like diabetes. Thus, it is highly suggested to promptly assess depression in chronic diseases like DM^14^.

Previous studies in Ethiopia showed inconsistent results in depression prevalence and associated factors among people living with diabetes. Additionally, the majority of previous studies conducted in Ethiopia were single-centre studies^15–17^. Hence, the current study was a multicentre and aimed to determine the magnitude of depression and its associated factors among patients living with diabetes attending outpatient clinic of Buno Bedele hospitals, Southwest Ethiopia.

## Methods

### Study area and period

The current study was done at an outpatient clinic of hospitals in Buno Bedele zone, namely Bedele hospital, Didessa hospital, and Chora hospital. Buno Bedele zone is found at 480 km away from Addis Ababa, the capital city of Ethiopia. It has an estimate of 841,158 total populations. These hospitals are providing health services which cover outpatient services, inpatient services, and emergency interventions. According to the data from reporting office, the outpatient departments are providing services for approximately 55,980, 11,246, and 5308 outpatient attendances per year in Bedele, Didesa, and Chora hospital, respectively. The study was performed from April to May 2021.

### Study design

A multicentre cross-sectional study was employed.

### Population

All adult diabetic patients attending follow-up care at outpatient clinics of public hospitals in Buno Bedele zone were the source population. All adult diabetic patients who visited the selected hospitals during the time of data collection and met the inclusion requirements made up the study population.

### Eligibility criteria

Diabetic patients with age at least 18 years old and attending follow-up at the selected hospitals for at least 6 months were included in the study. Critically ill patients who couldn’t respond were excluded from the study.

### Sample size determination and sampling technique

The sample size was calculated using a single population proportion formula.

$$\frac{{n = \left( {Z_{{\frac{\alpha }{2}}} } \right)^{2} \left( p \right)\left( q \right)}}{{d^{2} }}$$

where n = the desirable sample size, Z = 1.96 (the critical value at 95% level of significance). The expected prevalence of depression among diabetic patients attending hospitals in Buno Bedele was 50.0% since no previous study done in study area, d = 0.05 (level of precision/ acceptable marginal error) and q = 1 − p.

$${\text{n}} = \frac{{ (1.96)^{2} \left( {0.5} \right)\left( {0.5} \right) }}{{(0.05)^{2} }} \approx { 384}$$

Since the source population is less than 10,000 a finite population correction formula is applied to determine the final sample size.

$${\text{n}}^{\prime } = \frac{ ni }{{1 + n/N}} = \frac{384}{{1 + 384/1013}} = { 384}/{1}.{379 } = { 278}.{46 } \approx { 279}$$

Then, 10% of non-response was added.

Thus, nf = $\frac{{{\text{ n}}^{\prime } }}{{1 - {\text{\% non }}\,{\text{response}}}}$ = 279/0.9 = 310 was included in the study.

### Sampling technique

**Figure 1:**
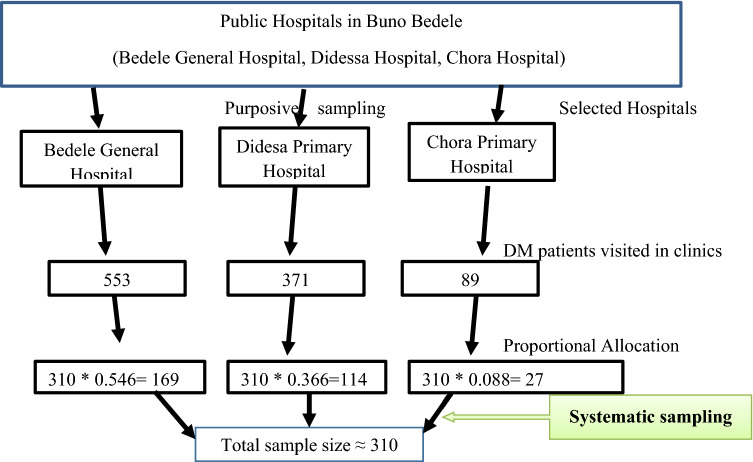
Schematic diagram showing sampling procedure for depression among diabetic patients attending follow-up at hospitals in Buno Bedele, Southwest Ethiopia, 2021.

A systematic random sampling technique was used to select the study participants. The hospitals’ medical records indicated that the total number of patients with diabetes who were on follow-up at the outpatient care of the hospitals were 1013. The sampling interval (k) was determined by dividing the total number of diabetic patients on follow-up at the hospitals to the calculated sample size (k = 1013/310  ≈  3). Because of the patients visit the hospital every one month or two months, all of them have chance to visit the hospital during data collection period. The first eligible participant was selected using a lottery method and the rest were included for every 3 intervals until the required sample size reached. The sample was proportionally allocated to each hospital (Fig. 1).

### Study variables

Depression was the dependent variable of the study. The independent variables were socio-demographic factors (age, sex, marital status, occupation, level of education, and place of residence), Behavioural and social support factors (alcohol consumption, cigarette smoking, khat chewing, physical activity, living condition, and social support) and clinical characteristics (duration of diabetes, comorbidity, type of diabetes, family history of diabetes, and family history of depression).

### Measurement of depression

A structured patient health questionnaire 9 (PHQ-9 items) was used to measure depression among adults living with DM. PHQ-9 is a nine items tool and for every item, there is a value set from 0 to 3, with a total range of 0–27 response scale. The value is 0 (not at all), 1 (several days), 2 (more than half of the days) or 3 (nearly every day). By adding these values, the study participants was considered as depressed if the PHQ-9 score is greater than or equal to 10^18^. The PHQ-9 appears to be a reliable and valid instrument that may be used to diagnose major depressive disorders among Ethiopian adults with sensitivity 86% and specificity 67% ^19^. Concerning the accuracy of PHQ-9 to diagnose depression, a cut-off score of 10 or above can be used regardless of age category^20^.

### Operational definition

*Depression* If patients with diabetes obtained the PHQ-9 score $\ge$ 10, the depression was diagnosed^18,21,22^.

*Current consumers* Patients who have consumed alcoholic beverage in the previous 12 months^23^.

*Previous consumers* Participants who have previously consumed alcohol but who have not done so in the previous 12 months^23^.

*Physically active* Those who are doing moderate to vigorous activity at work or leisure time for at least 30 min daily^24^.

*Social Support* The 3-item Oslo‐3 Social Support Scale (OSS‐3) was used to measure youth social support. The tool comprises valid values ranged from 3 to 14. A score ranging from 3 to 8 is classified as “poor support”, a score ranging from 9 to 11 classified as “moderate support” and a score ranging from 12 to 14 classified as “strong support”^25^.

*Comorbidity* Any chronic disease that coexisted with an index disease^26^.

### Data collection

#### Data collection instrument

The structured questionnaire was used to collect data. The questionnaire includes socio-demographic, behavioural characteristics, and clinical characteristics. Some parts of the questionnaire were adopted after reviewing similar related articles. The OSS-3 was used to assess the level of social support. The assessment of depression was done by administering PHQ -9. The data abstraction format was used to collect other disease-related data from their medical records.

#### Data collection process

The patients were interviewed to get data such as socio-demographic characteristics and about depression status. Disease-related data and medications were taken from patients’ medical records. Data collectors were two psychiatry nurses. One nurse and principal investigator were involved in the supervision of the data collection process.

#### Data quality assurance

The English version of the questionnaire was translated into Afan Oromo and translated back to English to check its consistency. The original and translated questionnaires were compared and the discrepancies were reviewed and resolved accordingly. The training was given for data collectors on the objective of the study, the contents of the questionnaires, and how to maintain the confidentiality and privacy of the study subjects. To check the functionality of the questionnaire and data extraction forms, a pre-test was carried out on 5% of the sample among diabetic patients at Mattu Karl hospital and necessary modifications were done. The collected data were checked for completeness and accuracy on spot before leaving the facilities.

#### Data processing and statistical analysis

Before data entry, data were coded and edited properly by the principal investigator. The data were entered into Epidata manager version 3.1 and double-entry verification was made and export to statistical package for social science (SPSS) version 24 for analysis. Descriptive statistics like frequency, and proportion were employed to describe the magnitude of depression, socio-demographic, clinical, and behavioural characteristics of patients. Bivariable logistic regression analysis was used to determine factors that candidates for multivariable logistic regression analysis. The variables found significant at *p* value < 0.25 in the bivariable analysis were included in multivariable logistic regression analysis. Multivariable logistic regression analysis was conducted to identify predictors of depression by controlling for confounders. Backward stepwise logistic regression variable selection method was employed and statistical significance was considered at *p* < 0.05.

### Ethics approval and consent to participate

The ethical clearance was obtained from Mattu University ethical committee. A permission to conduct the study was obtained from each hospital. All participants were informed of the purpose of the study and their participation was voluntary. Written informed consent was received from all participants. The name of the participant was omitted from the questionnaire; instead a medical record number was used to ensure confidentiality. This study was conducted based on the 1964 Helsinki declaration and its later amendments or comparable ethical standards.

## Results

### Socio-demographic characteristics of the study of participants

A total of 310 study participants were enrolled in this study, and the response rate was 100%. Male participants were counted for 161 (51.9%). The mean age of the participants was 47.22 years, and 127 (41.0%) had age range 41 to 60 years. The majority of the participants (85.2%) were married. In terms of respondents' educational status, 106 (34.2%) respondents had no formal education, while 109 (35.2%) attended primary school. About 149 (48.1%) of participants were farmers, and 156 (50.3%) of them were inhabitants of an urban area (Table 1).

**Table 1:** Socio-demographic characteristics of the study participants, 2021.

Variable	Category	Frequency	%
Sex	Male	161	51.9
	Female	149	48.1
Age	18–40	111	35.8
	41–60	127	41.0
	> 60	72	23.2
Marital status	Married	264	85.2
	Single	35	11.3
	Widowed	9	2.9
	Divorced	2	0.6
Educational status	No formal Education	106	34.2
	Primary school(1–8)	109	35.2
	Secondary school(9–12)	52	16.8
	College/University	43	13.9
Occupational status	Farmers	149	48.1
	Employees	52	16.8
	Private worker	55	17.7
	Merchant	25	8.1
	Students	18	5.8
	Other*	11	3.5
Residency	Urban	156	50.3
	Rural	154	49.7

*Students, and daily labourer.

### Behavioural characteristics, social support status, and clinical characteristics of the study participants

About 26 (8.4%) of the participants had history of cigarette smoking and only 4 (1.3%) of them were active smokers. Regarding alcohol use, 29 (9.4%) of the participants presently consume alcohol, and 83 (26.8%) of the participants had used alcohol in the past. In terms of chewing khat, 7 (2.3%) participants had previously chewed khat, and 71 (22.9%) were actively doing so.

About 65 (21.0%) participants had poor social support. In terms of living arrangements, 293 (94.5%) participants were living with their families. More than half (51.3%) of the participants had diabetes duration less than five years. A family history of depression was identified in 3.9% of the study participant. The magnitude of depression among the study participants was found to be 41.6% (Table 2).

**Table 2:** Behavioural characteristics, social support and clinical characteristics of the study participants, 2021.

Variable	Category	Frequency	%
Smoking status	Current smokers	4	1.3
	Previous smokers	26	8.4
	Never smoked	280	90.3
Current alcohol consumers	Yes	29	9.4
	No	281	90.6
Previous alcohol consumers	Yes	83	26.8
	No	227	73.2
Chew khat	Yes	71	22.9
	Yes, previously	7	2.3
	No, never	232	74.8
Physical activity	Yes	223	71.9
	No	87	28.1
Social support	Poor	65	21.0
	Moderate	163	52.6
	Strong	82	26.5
Living condition	Alone	16	5.2
	With family	293	94.5
	Camp	1	0.3
Type of DM	Type 1	63	20.3
	Type 2	247	79.7
Duration of DM (year)	< 5 years	159	51.3
	≥ 5 years	151	48.7
Presence of comorbidities	Yes	140	45.2
	No	170	54.8
Type of comorbidities	Hypertension	119	38.4
	Heart failure	44	14.2
	Asthma	24	7.7
	Ischemic heart disease	18	5.8
Number of comorbidity	1	85	60.7
	≥ 2	55	39.3
Family history of DM	Yes	74	23.9
	No	236	76.1
Family history of depression	Yes	12	3.9
	No	298	96.1
Depression	Yes	129	41.6
	No	181	58.4

### Factors associated with depression among the study participants

Bi-variable logistic regression analysis was done to examine the association of each independent variable with depression and screen for the multivariable model. Variables with a *p* value of less than 0.25 on bi-variable logistic regression entered into multivariable logistic regression.

Multivariable logistic regression analysis identified that females were 2.3 times more likely to have depression when compared with males [adjusted odds ratio (AOR) = 2.26, 95% confidence interval (CI) (1.30, 3.95)]. Similarly, participants with a duration of DM greater than 5 years were also 2.7 times more likely to have depression when compared to patients with less than 5 years of DM duration [AOR = 2.68, 95% CI (1.57, 4.56)].

When compared to patients with good social support, study participants with poor social support were 2.5 times more likely to experience depression [AOR = 2.46, 95% CI (1.10, 5.49)], and participants with moderate social support were 2.6 times more likely to have depression (AOR = 2.63, 95% CI (1.34, 5.16).

In comparison to participants who did not consume alcohol, those who did were 3.6 times more likely to experience depression [AOR = 3.55, 95% CI (1.20, 10.52)]. In addition, participants who had previously consumed alcohol had a 2.8-fold increased risk of having depression compared to those who had not [AOR = 2.81, 95% CI (1.40, 5.60)] (Table 3).

**Table 3:** Bi-variable and multivariable analysis of factors associated with depression among study participants, 2021.

Variables	Category	Depression		Bi-variable analysis	Multivariable analysis	*p* value
		Yes	No	COR(95% CI)	AOR (95% CI)	
Sex	Male	58	103	1	1	
	Female	71	78	1.62 (1.03, 2.55)	2.26 (1.30, 3.95)	0.004*
Age	18–40	33	78	1	1	
	41–60	51	76	1.59 (0.92, 2.72)	0.94 (0.49, 1.74)	0.852
	> 60	45	27	3.94 (2.10, 7.37)	1.67 (0.75, 3.68)	0.208
Educational status	Uneducated	55	51	1.50 (0.73, 3.00)	1.26 (0.56, 2.81)	0.576
	Primary school	45	64	0.98 (0.48, 1.90)	1.11 (0.50, 2.46)	0.793
	Secondary school	11	41	0.37 (0.15, 0.90)	0.44 (0.16, 1.18)	0.102
	College/University	18	25	1	1	
Duration of diabetes	< 5	47	112	1	1	
	≥ 5	82	69	2.83 (1.77, 4.52)	2.68 (1.57, 4.56)	< 0.001*
Social support	Poor (3–8)	33	32	2.81 (1.41, 5.60)	2.46 (1.10, 5.49)	0.028*
	Moderate (9–11)	74	89	2.27 (1.27, 4.00)	2.63 (1.34, 5.16)	0.005*
	Strong (12–14)	22	60	1		
Current alcohol consumption	Yes	23	6	6.33 (2.50, 16.00)	3.55 (1.20, 10.52)	0.023*
	No	106	175	1	1	
Previous Alcohol consumption	Yes	51	32	3.04 (1.81, 5.10)	2.81 (1.40, 5.60)	0.003*
	No	78	149	1	1	
Physical Activity	Yes	78	145	1	1	
	No	51	36	2.63 (1.59, 4.30)	1.68 (0.94, 3.00)	0.083

*Statistically significant at *p* value < 0.05, COR = crude odds ratio at 95% confidence interval; AOR = adjusted odds ratio at 95% confidence interval.

## Discussion

Depression is common in patients with DM. Despite this fact, there is a lack of attention to screening and early treatment of depression among diabetic patients. Identifying the prevalence and risk factors is helpful for early screening and treatment for these public health issues.

The present study revealed that the prevalence of depression among DM is 41.6% [95% CI (36.1, 47.1)]. This finding is in line with a study conducted in Southwest Ethiopia (37.0%)^27^, in Jimma University Specialized Hospital (43.6%)^16^, and Northwest Ethiopia (40.4%)^17^. However, the finding of current study is lower than finding of the study conducted in Rwanda which reported 83.8% of depression among participants^28^. The reason for this discrepancy could be the difference in socio-economic characteristics of the population. Environmental and political factors might also play a role in the discrepancy. Rwanda was a country where a terrible event of genocide occurred, which might have contributed to the higher prevalence of depression in the country.

The current study identified that depression was significantly associated with sex. Female participants were more likely to have depression when compared to male participants. Similarly, a study conducted in Rwanda, Northwest Ethiopia, and Hawassa University Comprehensive Specialized Hospital reported that female participants were more likely to develop depression^15,28–30^. A higher magnitude of depression among female participants might be due to different factors of social determinants such as educational and job opportunities, living wages, healthful foods, social norms and attitudes, and gender discrimination. Sexual and domestic violence could also contribute to the higher risk in females^31,32^. Additionally, changes in sex hormone go through at different times of females’ lives may play a role, and make them vulnerable to depression. Females are more likely to experience depression due to hormonal changes beginning in adolescence and continuing into menopause. This risk is further increased by the fact that the most extreme hormonal swings take place during perimenopause^33^.

This study showed that participants with longer duration of DM were more likelihood to have depression. This finding is consistent with previous study conducted in Ethiopia which indicated that patients with longer duration of DM were more likely to have depression compared with those lived with short duration^27^. The increased risk of depression with the duration of the illness could be because increased chance of developing DM complication as well as comorbidities as DM duration is higher. Diabetic patients require ongoing self-medical care on daily basis which limits their usual activities and causes impairments of their normal daily activities which are a risk to develop depression over time^34^.

This study indicated that participants with poor social support and moderate social supports were more likely to have depression compared to patients with good social support. This finding is in line with the study conducted at Hawassa University Comprehensive Specialized Hospital, Southern Ethiopia which reported participants with no social support were more likely to have depression when compared to good social support^15^. The justification could be patients who had poor social support are influenced by negative life stressors and it also plays a crucial role in the disturbance of the coping process for those with DM. Thus, poor social support levels are an important risk factor for the subsequent development or worsening of depression.

Regarding the behavioural characteristics of the study participants, this study revealed that current and previous alcohol consumers were more likely to have depression compared to those who were not consume alcohol. This study is consistent with the studies conducted in different settings that revealed depression was most strongly associated with current and previous alcohol consumption. Accordingly, a study conducted in Hawassa University Comprehensive Specialized Hospital showed that one of the potential predictors of depression was alcohol consumption^15^. A national health survey also indicated that alcohol consumers were more likely to have depression when compared to their counterparts^35^. This might be because of the withdrawal effect; the lack of dopamine and diminished receptors can lead to feelings of sadness and hopelessness^36^. In addition, alcohol consumption remains controversial as many scientific studies suggest that alcohol had a high risk than benefit. Risks like glucose intolerance lead to weight gain, impairs adherence to DM self-care and management.

The cross-sectional nature of the study may not allow showing the temporal relationship. It was impossible to know the direction of cause and effect relationship of associated factors of depression in DM patients. Some of independent variables such as alcohol consumption and khat chewing were not measured by the gold standard methods. Because the data were gathered through face-to-face interviews, this study may be prone to social desirability bias. Moreover, we didn’t utilize any other clinical criteria to definitively identify depression; instead, we just employed the PHQ-9 questionnaire to assess the depression. Finally, this study assessed depression for only diabetic patients being treated and on follow-up at primary and secondary care settings and may not be generalized to all diabetes patients.

## Conclusion and recommendation

According to this study, depression is relatively common among people living with diabetes. Depression is significantly associated with female gender, having long duration of DM, having poor social support, and current and previous history of alcohol consumption. Healthcare professionals should consider screening for depression using the PHQ-9 or other validated tools in all diabetic patients, especially in those who are at higher risk. Additionally, educate patients about risk of alcohol consumption on depression is important to tackle alcohol-induced depression. Researchers should conduct further investigation regarding the factors contributing to depression among patients with chronic illnesses, especially diabetes using strong study design.

## Data Availability

The datasets used and/or analyzed during the current study are available from the corresponding author on reasonable request.

## Abbreviations

DM: Diabetes mellitus
OSS‐3: Oslo‐3 social support scale
SPSS: Statistical package for social science
PHQ-9: Patient health questionnaire-9
AOR: Adjusted odds ratio
COR: Crude odds ratio
